# Apolipoprotein E deficiency and high-fat diet cooperate to trigger lipidosis and inflammation in the lung via the toll-like receptor 4 pathway

**DOI:** 10.3892/mmr.2015.3774

**Published:** 2015-05-12

**Authors:** QIUFANG OUYANG, ZIYANG HUANG, HUILI LIN, JINGQIN NI, HUIXIA LU, XIAOQING CHEN, ZHENHUA WANG, LING LIN

**Affiliations:** 1 Cardiovascular Department, The Second Affiliated Hospital and Second Clinical Medical College, Fujian Medical University, Quanzhou, Fujian 362000, P.R. China; 2 Key Laboratory of Cardiovascular Remodeling and Function Research, Chinese Ministry of Education and Chinese Ministry of Health, Shandong University Qilu Hospital, Jinan, Shandong 250012, P.R. China; 3 Rheumatism Department, The Second Affiliated Hospital and Second Clinical Medical College, Fujian Medical University, Quanzhou, Fujian 362000, P.R. China

**Keywords:** apolipoprotein E, diet, toll-like receptor, inflammation, lipid

## Abstract

Apolipoprotein E deficiency (ApoE^−/−^) combined with a high-fat Western-type diet (WD) is known to activate the toll-like receptor (TLR4) pathway and promote atherosclerosis. However, to date, the pathogenic effects of these conditions on the lung have not been extensively studied. Therefore, the present study examined the effects of ApoE^−/−^ and a WD on lung injury and investigated the underlying mechanisms. ApoE^−/−^ and wild-type mice were fed a WD or normal chow diet for 4, 12 and 24 weeks. Lung inflammation, lung cholesterol content and cytokines profiles in bronchoalveolar lavage fluid (BALF) were determined. TLR4 and its main downstream molecules were analyzed with western blot analysis. In addition, the role of the TLR4 pathway was further validated using TLR4-targeted gene silencing. The results showed that ApoE^−/−^ mice developed lung lipidosis following 12 weeks of receiving a WD, as evidenced by an increased lung cholesterol content. Moreover, dependent on the time period of receiving the diet, those mice exhibited pulmonary inflammation, which was manifested by initial leukocyte recruitment (at 4 weeks), by increased alveolar septal thickness and mean linear intercept as well as elevated production of inflammation mediators (at 12 weeks), and by granuloma formation (at 24 weeks). The expression levels of TLR4, myeloid differentiation primary response 88 (MyD88) and nuclear factor kappa B were markedly upregulated in ApoE^−/−^ WD mice at week 12. However, these effects were ameliorated by shRNA-mediated knockdown of TLR4. By contrast, ApoE^−/−^ ND or wild-type WD mice exhibited low-grade or no inflammation and mild lipidosis. The levels of TLR4 and MyD88 in those mice showed only minor changes. In conclusion, ApoE deficiency acts synergistically with a WD to trigger lung lipidosis and inflammation at least in part via TLR4 signaling.

## Introduction

There is compelling evidence that apolipoprotein E deficiency (ApoE^−/−^) combined with a high-fat diet regulates toll-like receptor 4 (TLR4) expression and promotes atherosclerosis development. Massaro and Massaro (1) reported that ApoE^−/−^ mice displayed reduced alveologenesis as compared with wild-type strain controls, and that ApoE^−/−^ had an effect on lung pathological changes. Similarly, Naura *et al* (2) demonstrated that ApoE^−/−^ mice on a high-fat diet displayed lung inflammation. Goldklang *et al* (3) indicated that ApoE^−/−^ mice on a high-fat Western-type diet (WD) showed emphysema due to TLR4 activation (3). Samokhin *et al* (4) suggested that ApoE^−/−^ mice on a high-fat diet developed granulomas similar to those observed in human sarcoidosis. Accordingly, reports of the effects of HFD on lung pathological changes in ApoE^−/−^ mice differ greatly.

Macrophages are important inflammatory cells implicated in the initiation of inflammation, and they have critical roles in the pathogenesis of foam cell formation (5). TLR4 is a key initiator of innate immunity that is able to promote an adaptive immune response. TLR4 recognizes lipopolysaccharide (LPS), resulting in the activation of the myeloid differential factor 88 (MyD88)- and toll-interleukin-1 receptor domain-containing adapter inducing interferon-β (TRIF)-dependent downstream signaling pathways. TLR4 signalling has a critical role in the progression of atherosclerosis and lung inflammation (6,7). A previous study by our group revealed that ApoE^−/−^ mice developed pulmonary capillaritis via up-regulation of TLR4 and nuclear factor (NF)-κB (8). However, to date, the role of TLR4 signalling in the pathogenesis of lung lipidosis have not been studied, to the best of our knowledge.

The aim of the present study was to determine whether ApoE deletion combined with hypercholesterolemia induces lung inflammation and lipidosis. Furthermore, TLR4 knockdown was employed to investigate whether TLR4 signalling is implicated in those pathological changes.

## Materials and methods

### Animals and experimental design

All of the procedures and protocols were approved by the Animal Care Committee of Fujian Medical University (Quanzhou, China) and followed the guidelines of the Animal Management Rules of the Chinese Ministry of Health. Eighty eight-week-old male ApoE^−/−^ mice and sixty age- and gender-matched wild-type mice with a C57BL/6 genetic background (B6) were obtained from the Peking University Animal Centre (Beijing, China). In the first group, thirty ApoE^−/−^ and B6 mice were fed a WD (containing 0.25% cholesterol and 15% cocoa butter; MD12032) or a normal chow diet (ND; MD12031; Yangzhou Medicience Ltd, Yangzhou, China) for 4, 12 or 24 weeks, respectively (n=10 in each group). In the second group, the ApoE^−/−^ and wild-type mice were injected with short hairpin TLR4 interference lentivirus (Lv-shTLR4) or empty vector (both from Invitrogen Life Technologies, Paisley, UK) at 1×10^8^ transducing units for each mouse through the caudal vein (n=10). They were fed the WD for 12 weeks. All the animals were under standardized lighting conditions (12-h light/dark cycle) and temperature (21±1°C). Mineral water was administered *ad libitum*. At the end of the experiment the mice were sacrificed by overdose of pentobarbital (90 mg/kg; intraperitoneal injection; Huayehuanyu Ltd, Beijing, China). The bronchoalveolar lavage fluid (BALF) was collected and the left lung lobe tissue was collected for histomorphological examination, whereas the right lung lobe tissue was collected for RNA and protein analysis.

### Hematoxylin and eosin (HE) staining for lung pathomorphological changes

The lung tissue was stained with hematoxylin and eosin (HE; Sigma-Aldrich, St. Louis, MO, USA). The mean linear intercept and septal thickness were quantified as described by Wendel *et al* (9). This assessment was repeated for 10 terminal respiratory units in one random tissue section per mouse. All the images were acquired using a BX51 microscope (Olympus, Center Valley, PA, USA) and analyzed using Image-Pro Plus 6.0 (Media Cybernetics, Inc., Bethesda, MD, USA). The evaluation was performed by two experienced pathologists who were blinded to the treatments that the mice had received, according to methods previously described (10).

### Oil red O staining for lipidosis in the lung and quantitation of pulmonary cholesterol content

For assessment of lipidosis, the frozen lung sections were stained with Oil Red O (Sigma-Aldrich). The cholesterol content of the lung tissue was quantified according to Bates *et al* (11). The free and total cholesterol contents were calculated using a cholesterol standard (Sigma-Aldrich). The cholesteryl ester content was calculated by subtracting the free cholesterol from the total cholesterol for each sample. The cholesterol content was expressed as ‘micrograms of lipid per gram of animal’.

### Double immunofluorescent staining for assessment of TLR4 in macrophages

For the localization of TLR4 expression in macrophages, double immunofluorescence staining using the CD68 macrophage marker and anti-TLR4 (all diluted at 1:100; Abcam, Cambridge, UK) was performed on the lung sections. CD68 and TLR4 double-labelled cells were quantified as a fraction of the total cell nuclei in each lung section.

### Western blot analysis of TLR4, MyD88, NF-κB p65, TRIF and interferon regulatory factor 3 (IRF3) expression in the lung tissues

To determine the TLR4, MyD88 phosphorylated (p)-NF-κB, TRIF3 and IRF3 protein levels in the lung tissue, western blot analysis was performed as described by Zhang *et al* (12). Primary antibodies (all from Abcam, Cambridge, UK) were used at the indicated dilutions as follows: Rabbit polyclonal to TLR4 (cat. no. ab13556; 1:500); rabbit polyclonal to MyD88 (cat. no. ab2064; 1:1,000); rabbit polyclonal to NF-κB p65 (cat. no. ab7970; 1:1,000); rabbit polyclonal to p65-NF-κB (cat. no. ab7970; 1:500); rabbit polyclonal to TRIF (cat. no. ab13810; 1:500); rabbit monoclonal to IRF3 (cat. no. ab68481; 1:1000). Proteins (30 *µ*g) were run on a 10% SDS-PAGE gel and transferred to polyvinylidene fluoride membranes. Following incubation with 10% non-fat milk for 1 h, the membranes were probed with primary antibodies overnight at 4°C and then incubated with HRP-labeled goat anti-rabbit secondary antibodies (1:2,000; Santa Cruz Biotechnology, Inc., Dallas, TX, USA). The protein levels were normalized using β-actin as a loading control. The relative optical density of the protein bands was measured using a Zeineh Laser Densitometer (Biomed Instruments, Inc., Fullerton, CA, USA) after subtracting the film background.

### ELISA of cytokine profiles in BALF

The cytokine concentrations were determined using sandwich ELISA for interferon-γ (IFN-γ), tumor necrosis factor alpha (TNF-α), interleukin-4 (IL-4), IL-6 and IL-17 (IFN-γ, TNF-α, IL-4, IL-6 and IL-17 ELISA kits all from eBioscience, San Diego, CA, USA) in BALF according to the manufacturer’s instructions. All the measurements were performed in duplicate.

### Serum lipid analysis

The fasting serum samples were collected in 20-week-old mice of different genotypes following fasting for 8 h. The total cholesterol (TC), triglycerides (TG), high-density lipoprotein cholesterol (HDL-C) and non-HDL-C were measured as previously described (13) using reagents from Nanjing KeyGen Biotech Co., Ltd. (Nanjing, China).

### Lentiviral short hairpin RNA (Lv-shRNA)-mediated TLR4 gene silencing

The shRNA targeting of the TLR4 gene (GenBank accession no., NM021297.2) was screened and tested according to the protocol of according to Zhu *et al* (14). The target sequence was designed and chemically synthesized by the United Gene Company (Shanghai, China). This shRNA comprises an RNA duplex containing a sense strand: 5′-GATCCGCACTCTTGATTGC AGTTTCATTCAAGAGATGAAACTGCAATCAAGAGTG CTTTTTTG-3′ and an antisense strand: 5′-AATTCAAA AAAGCACTCTTGATTGCAGTTTCATCTCTTGAATGA AACTGCAATCAAGAGTGCG-3′. The inserted TLR4 cDNA sequence was confirmed by DNA sequencing.

### Reverse transcription quantitative polymerase chain reaction (RT-qPCR)

qPCR was performed to test the efficacy of TLR4 knockdown. Total RNA was isolated from the lung tissue using the RNAiso Plus kit (Takara Biotechnology Co., Ltd., Dalian, China). A total of 500 ng RNA was used as the template for cDNA generation with the RNA RT kit (Takara Bio, Inc., Shiga, Japan). cDNA was immediately reverse-transcribed from the isolated RNA and subsequently qPCR was performed using Power SYBR Green PCR Master Mix (Takara Bio, Inc.) on the Master Mix System (Roche, Basel, Switzerland). The primer sequences (5′ to 3′) were TLR4 forward, ATGGCATGGCTTACACCACC and reverse, GAGGCCAATTTTGTCTCCACA; GADPH forward, AGGTCGGTGTGAACGGATTTG and reverse, TGTAGACCATGTAGTTGAGGTCA. The PCR conditions were as follows: Initial denaturation at 95°C for 5 min, denaturation at 94°C for 45 sec, annealing at 50°C for 1 min and extension at 72°C for 1 min. The PCR was performed for 35 cycles followed by a final extension step at 72°C for 10 min. The PCR product was quantitatively analyzed with LabWorks 4.5 analysis software (UVP LLC, Upland, CA, USA). Relative quantification of the target gene mRNA was performed using the comparative ΔΔCT-method, normalized to (GADPH) and a relevant ApoE^−/−^ empty vector control to obtain 2^−ΔΔCT^.

### Statistical analysis

All values are expressed as the mean ± standard error unless otherwise indicated. The group comparisons were performed using Student’s *t* test (2-sample test) or analysis of variance. A P-value of 0.05 was regarded to indicate a statistically significant difference between values. The statistical analysis was performed using SPSS 17.0 software (SPSS, Chicago, IL, USA).

## Results

### Age-dependent inflammation and lipidosis in the lungs of ApoE^−/−^ WD mice

**Figure 1:**
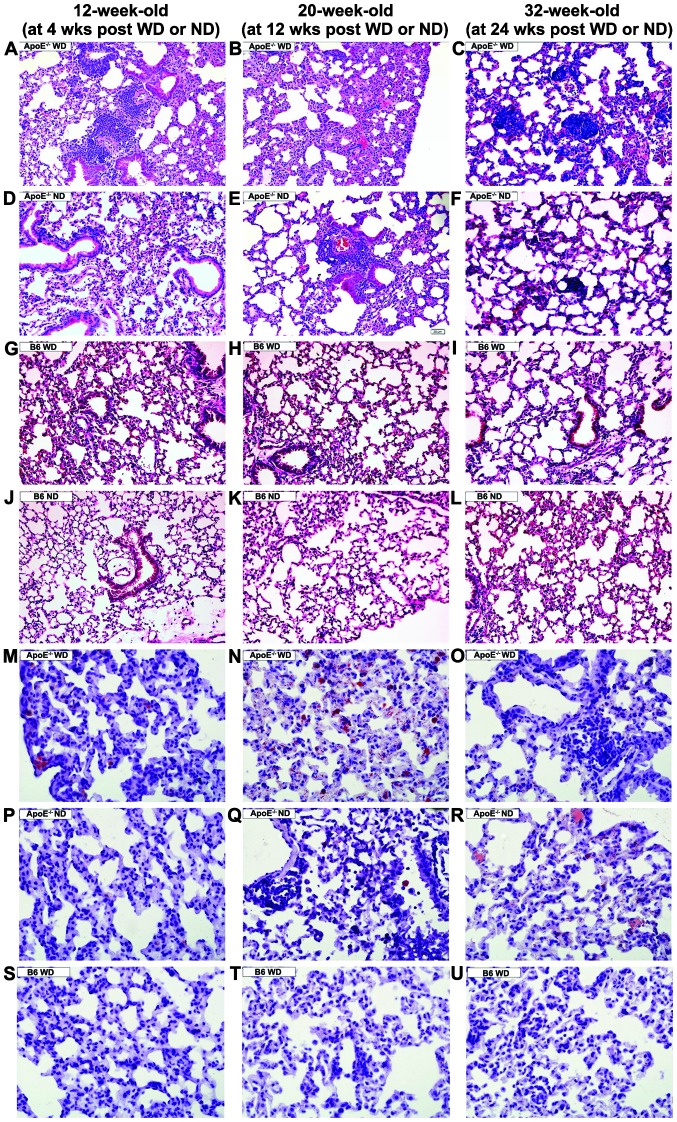

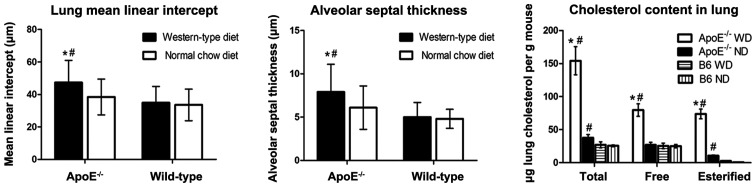
Lung inflammation and lipidosis in mice of different genotypes fed an ND or WD. Micrography demonstrating (A) increased inflammatory cell infiltration, (B and E) exudation and thickened alveolar septa, and (C and F) granuloma development in the ApoE^−/−^ mice. (D, G–L, S–U) Normal lung structure was present. (M–R) Oil red O staining indicated that foam cells (red) were present in the septa and alveolar lumina. A–L: hematoxylin and eosin staining; magnification, ×200; M–U: Oil red O staining, magnification, ×400. Bars represent the mean ± standard error of seven mice. ^*^P<0.05 compared with mice of the same genotype on a ND. ^#^P<0.05 compared with the B6 mice fed on the same diet. B6, C57BL/6J; ND, normal chow diet; WD, high-fat Western-type diet; ApoE, apolipoprotein.

In the ApoE^−/−^ mice receiving the WD for four weeks, inflammatory cell infiltration was noted around the capillaries and venules (Fig. 1A). Following WD for 12 weeks, the ApoE^−/−^ mice showed thickened alveolar septa, exudate-filled alveolar spaces, ruptured septa and bullae formation (Fig. 1B). Widely distributed granulomas were observed in the ApoE^−/−^ mice following WD for 24 weeks (Fig. 1C). By contrast, among the animals receiving ND treatment for 4 or 12 weeks, the ApoE^−/−^ mice displayed a minimal number of inflammatory cells in the peribronchiolar and perivascular sites (Fig. 1D and 1E), and very few granulomas developed at 24 weeks of ND (Fig. 1F). Those manifestations were absent in the ApoE^−/−^ mice fed an ND for four weeks or in the wild-type mice fed a WD for 24 weeks (Fig. 1G–L). Collectively, these data suggested that pulmonary inflammation developed more extensively and earlier in the ApoE^−/−^ WD mice than in the littermates on ND. In the wild-type mice on WD, no signs of inflammation were observed, as indicated by normal bronchioles and alveoli.

Lipid-laden cells were observed by oil red O staining in the septa and alveolar lumina in the ApoE^−/−^ WD mice at 12 weeks (Fig. 1N), whereas scattered lipid-filled cells were observed in the ApoE^−/−^ WD mice at 4 and 24 weeks (Fig. 1M and O) or the ApoE^−/−^ ND littermates at 24 weeks. No foam cells were present in the wild-type mice on the WD even for 24 weeks (Fig. 1S–U).

Additionally, at 12 weeks, the alveolar septal thickness and mean linear intercept were obviously greater in the ApoE^−/−^ WD mice when compared with that in the ApoE^−/−^ ND or B6 WD mice. The ApoE^−/−^ ND mice showed a marginal increase in alveolar septal thickness and mean linear intercept; however, with no statistical significance. The B6 WD mice exhibited normal alveoli and septal thickness.

The lung cholesteryl content quantitation showed that at 12 weeks, the total cholesterol content in the lungs of ApoE^−/−^ WD mice was elevated 5.69-fold relative to that in the B6 WD mice and 4.08-fold compared to that in the ApoE^−/−^ ND mice, predominantly due to marked elevation in cholesteryl ester levels. In ApoE^−/−^ WD mice, cholesteryl ester was 29.48-fold higher than that in the B6 WD mice and 6.95-fold higher than that in the ApoE^−/−^ ND mice. In the wild-type B6 mice, the levels of lung cholesterol were altered insignificantly, irrespective of the diet.

### Increased TLR4 expression in pulmonary macrophages of ApoE^−/−^ WD mice

**Figure 2:**
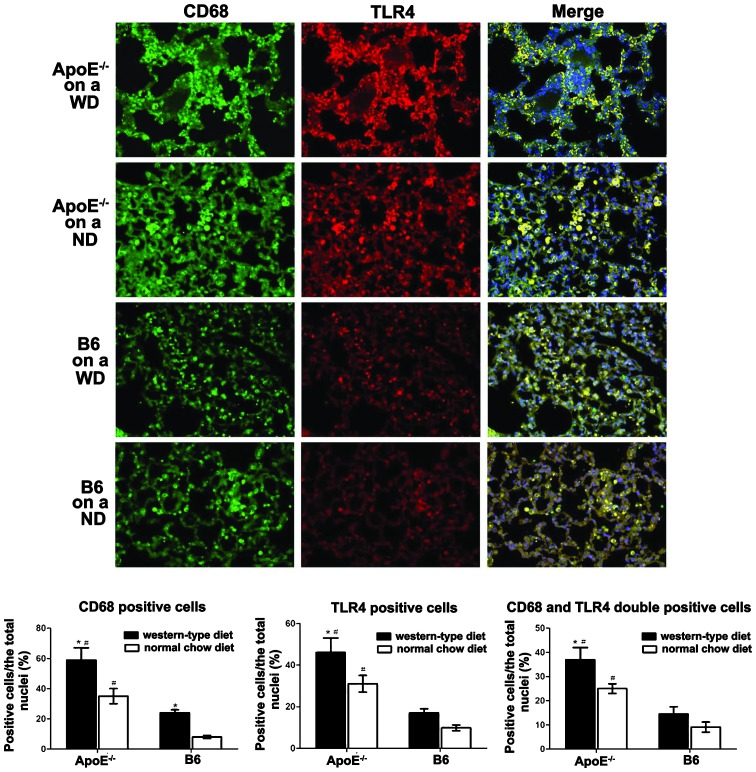
Double-label immunofluorescence of lung sections with macrophage CD68 (green) and TLR4 (red) markers. The nuclei were stained with DAPI (blue) (magnification, x200). Increased CD68^+^ TLR4^+^ cells in the ApoE^−/−^ mice fed on WD or ND for 12 weeks. The bars represent the mean ± standard error of seven mice. ^*^P<0.05 compared to the same genotype mice fed on 12-week ND. ^#^P<0.05 compared with the B6 mice fed on the same diet. B6, C57BL/6J; TLR4, toll-like receptor 4; ND, normal chow diet; WD, high-fat western-type diet; Apo, apolipoprotein.

TLR4 in macrophages is critical in pulmonary inflammation and lipidosis. To investigate the implications of TLR4 in macrophages in the pathogenesis of lung injury, TLR4 expression and lung macrophages were co-localised with double immunofluorescent staining. The CD68^+^ macrophages (green) in the lung were enriched in TLR4 (red). The ApoE^−/−^ mice fed a WD for 12 weeks exhibited markedly greater macrophage infiltration and TLR4 expression in the alveolar septum compared with that in the B6 WD mice (Fig. 2). The ApoE^−/−^ ND mice displayed a moderately increased number of CD68^+^ TLR4^+^ cells (yellow) in the lung (Fig. 2).

### TLR4 and its downstream activation in ApoE^−/−^ WD mice

**Figure 3:**
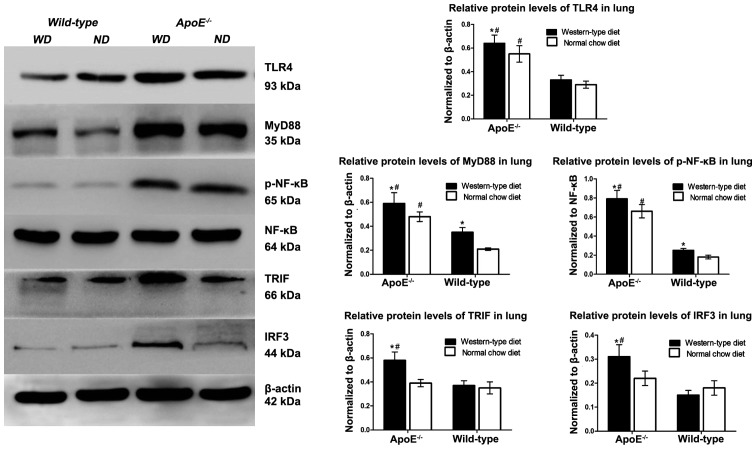
Expression of TLR4 and its major downstream molecules in lung tissue as determined by western blotting following 12-week WD or ND. β-actin or NF-κB served as the loading control. The bars represent the mean ± standard error of four separate experiments. ^*^P<0.05 compared with mice of the same genotype fed on the ND. ^#^P<0.05 compared with the B6 mice fed on the same diet. TLR4, toll-like receptor 4; ND, normal chow diet; WD, high-fat Western-type diet; MyD88, myeloid differentiation protein 88; NF-κB, nuclear factor-kappa B; p-NF-κB, phosphorylated NF-κB; TRIF, TIR-domain-containing adapter-inducing interferon-β; IRF3, interferon regulatory factor 3; Apo, apolipoprotein.

MyD88/NF-κB and TRIF/IRF3 are key downstream molecules in the TLR4 signalling pathway. To determine whether these molecules were involved in pulmonary inflammation and lipidosis, the protein levels of TLR4, as well as its downstream MyD88-dependent (MyD88, NF-κB) and -independent (TRIF and IRF3) molecules in the lungs were detected by western blot analysis. Following WD for 12 weeks, the levels of TLR4, MyD88 and p-NF-κB were markedly upregulated in the ApoE^−/−^ mice compared with those in the B6 mice. The TRIF and IRF3 levels were significantly increased (Fig. 3). Among the mice receiving the ND, the levels of MyD88 and NF-κB in the ApoE^−/−^ mice were moderately elevated relative to those in the corresponding B6 controls; however, the expression levels of TRIF and IRF3 were marginally altered in the ApoE^−/−^ mice in comparison with those in the B6 animals.

### Increased BALF levels of IFN-γ, TNF-α, IL-4, IL-6 and IL-17 in ApoE^−/−^ WD mice

**Figure 4:**
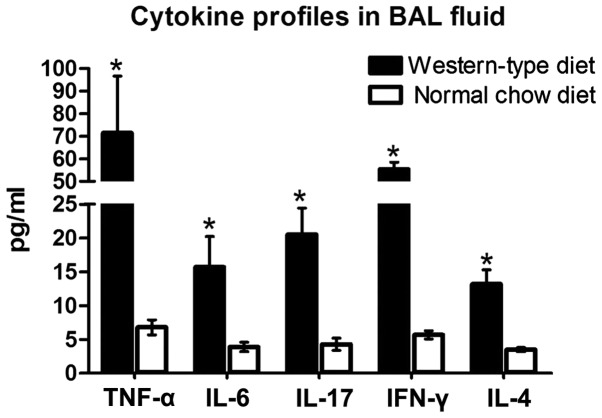
Cytokine level profile in BALF of mice following 12-week high-fat Western-type or normal chow diets assayed by ELISA. ^*^P<0.05 vs. the ApoE^−/−^ mice on a normal chow diet. The results are expressed as the mean ± standard error for seven mice. BALF, bronchoalveolar lavage fluid; TNF-α, tumour necrosis factor alpha; IL-6,17,4, interleukin-6,17,4; IFN-γ, interferon-γ.

An inflammatory response involves the recruitment of immune cells and changes in cytokines. The levels of IFN-γ, TNF-α, IL-4. IL-6 and IL-17 in BALF were detected in the mice following 12-weeks of reciving their respective diet. In the ApoE^−/−^ mice, the levels of pro-inflammatory cytokines IFN-γ, TNF-α, IL-6 and IL-17 in BALF were markedly elevated by 8.7-fold, 9.5-fold, 3.0-fold and 3.8-fold, respectively, compared with those in the littermates on an ND. The anti-inflammatory cytokine IL-4 was significantly increased by 2.7-fold (Fig. 4). The levels of these cytokines were undetectable in the B6 mice receiving ND or WD.

### Hyperlipidemia in the ApoE^−/−^ WD mice

The serum lipids were detected to evaluate the effects of the high-fat Western-type diet on the lipid profiles of the experimental mice. Compared with those of their corresponding genotype mice receiving the ND, the serum TC, TG and non-HDL-C levels were markedly elevated in the ApoE^−/−^ mice receiving the WD, and, to a lesser extent, in the wild-type mice following 12 weeks of WD. The HDL-C levels in the ApoE^−/−^ mice decreased, whereas they remained comparable in the wild-type mice (Table I).

### TLR4-targeted gene silencing ameliorates inflammation and lipidosis in the ApoE^−/−^ mice

**Figure 5:**
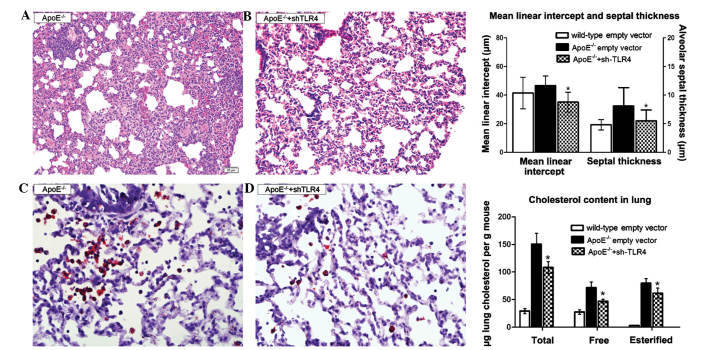
Effects of TLR4 interference on lung inflammation and lipidosis in the ApoE^−/−^ mice fed on a high-fat Western-type diet for 12 weeks. (A and B) Hematoxylin and eosin staining illustrated that relative to the ApoE^−/−^ empty vector controls A, the ApoE^−/−^ sh-TLR4 mice B showed ameliorated cell infiltration, exudation, reduced septal thickness and a mean linear intercept. (C and D) Concurrently, TLR4 interference attenuated the foam cells in the lung as well as the lipid content. A and B, hematoxylin and eosin; C and D, Oil red O staining (magnification, x200). The histograms represent the mean ± standard error of seven mice. ^*^P<0.05 compared with the ApoE^−/−^ empty vector mice. sh-TLR4, short hairpin toll-like receptor 4-targeted gene silencing; Apo, apolipoprotein.

To further validate whether the TLR4 molecule contributed to lung injury in the ApoE^−/−^ mice, the TLR4 pathway was blocked using Lv-shRNA-TLR4. Treatment with Lv-TLR4 shRNA for 12 weeks significantly attenuated the septal thickness (5.5±1.9 *µ*m vs. 8.1±3.2 *µ*m) and the mean linear intercept (35.1±6.9 *µ*m vs. 46.7±11.6 *µ*m). The lung cholesterol content, including the esterified and free cholesterol, was diminished (Fig 5). The serum lipid profiles changed insignificantly with the TLR4-shRNA lentivirus treatment (data not shown).

### Inactivating the MyD88-dependent NF-κB downstream pathways by TLR4 interference

**Figure 6:**
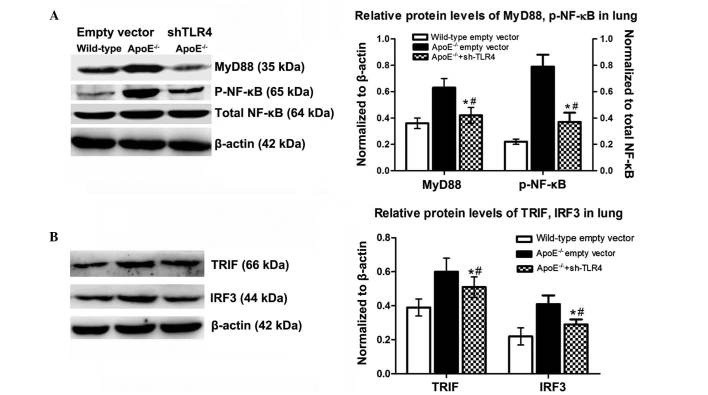
Effects of TLR4 interference on (A) MyD88, p-NF-κB, (B) TRIF and IRF3 expression in the lung of mice following 12 weeks of a high-fat Western-type diet as determined by western blotting. β-actin or total NF-κB served as a loading control. Bars represent the mean ± standard error of three independent experiments with similar results. ^*^P<0.05 compared to ApoE^−/−^ empty vector mice. ^#^P<0.05 compared with wild-type mice. MyD88, myeloid differentiation protein 88; NF-κB, nuclear factor-kappa B; P-NF-κB, phosphorylated NF-κB; TRIF, TIR-domain-containing adapter-inducing interferon-β; IRF3, interferon regulatory factor 3; Apo, apolipoprotein.

Following TLR4-targeted gene silencing, all the signalling molecules (MyD88, p-NF-κB, TRIF, IRF3) were downregulated by 46, 53, 15, and 29%, respectively, with a predominant inhibitory effect on the MyD88-dependent pathway. In the ApoE^−/−^ mice, levels of these signaling molecules in the lung remained significantly higher than those of the B6 counterparts fed the WD (Fig. 6).

### Efficiency and safety of lentivirus transfection in vivo

**Figure 7:**
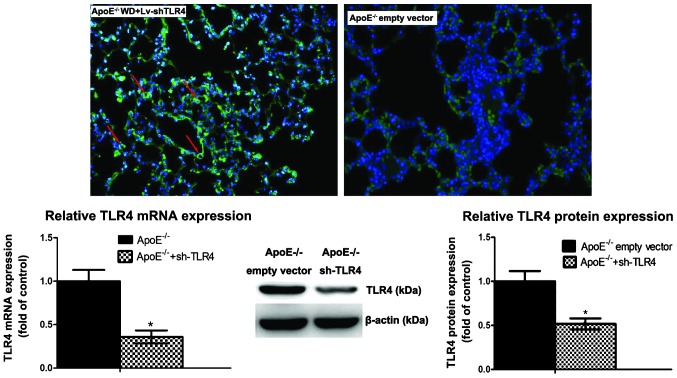
Efficiency of lentivirus transfection by monitoring GFP fluorescence, TLR4 mRNA and protein expression in the lung 12 weeks following lentivirus injection. Arrows indicate GFP^+^ cells. Original magnification, ×400. For TLR4 mRNA expression, the comparative threshold cycle method was used to analyze the gene expression normalized to GAPDH by polymerase chain reaction analysis. TLR4 protein expression was quantitated by western blotting. Bars represent the mean ± standard error from triplicate values. ^*^P<0.05 vs. control. GFP, green fluorescence protein; sh-TLR4, short hairpin toll-like receptor 4-targeted gene silencing; Apo, apolipoprotein.

GFP fluorescence in the lung was still observed at 12 weeks following transfection, which suggested a successful transfection of shRNA lentivirus (Fig. 7). To further confirm the efficacy of lentivirus-mediated TLR4 gene silencing, the levels of TLR4 mRNA and protein in the lung were determined. Compared with ApoE^−/−^ WD mice, TLR4 mRNA expression in the Lv-sh-TLR4 subgroup was reduced by 64.1% paralleled by a reduction of TLR4 protein by 49.3%. No adverse effects occurred during the trial, indicating that lentivirus transfection was safe (data not shown). Collectively, the results demonstrated an efficient and safe lentivirus-mediated transfection of shRNA *in vivo*.

## Discussion

The present study reported that in genetically susceptible ApoE^−/−^ mice, a 12-week high-fat diet induced pulmonary lipidosis, as illustrated by an elevated lung cholesterol content and increased alveolar macrophage foam cell formation. It was discovered that, dependent on the time period of receiving the diet, the ApoE^−/−^ WD mice exhibited inflammatory injury that was characterized by initial leukocyte recruitment (week 4), increased alveolar septal thickness, a mean linear intercept (week 12) and granuloma formation (week 24). The ApoE^−/−^ ND mice or wild-type WD mice manifested a low-grade or no inflammation. The expression of TLR4 and its downstream molecules MyD88, p-NF-κB, TRIF and IRF3 were markedly upregulated in the 12-week-old ApoE^−/−^ WD mice, whereas their expression was slightly changed in the ApoE^−/−^ ND and wild-type WD mice. Blocking the TLR4 pathway was able to ameliorate lipidosis and inflammation in the ApoE^−/−^ WD mice. To the best of our knowledge, the present study was the first to reveal that an ApoE deficiency combined with a high-fat diet caused lung lipidosis and inflammation via the TLR4 signaling pathway. Of note, it was found that the blocking of TLR4 could not fully ameliorate lipidosis and inflammation, suggesting that other signaling pathway(s) may be involved in those pathomorphological changes.

### Inflammatory response in ApoE^−/−^ WD mice

Evidence has indicated that the respiratory system and cardiovascular system are intricately intertwined (15). It has been well documented that the ApoE^−/−^ mice on a high-fat diet developed pulmonary arterial hypertension (16); those on a Paigen diet exhibited more severe pulmonary hypertension (17). An ApoE mimetic peptide was able to prevent airway inflammation and goblet cell hyperplasia in ApoE^−/−^ mice challenged by house dust mites (18). The present study indicated that ApoE^−/−^ WD mice developed lung inflammation, which was characterized by initial inflammatory cell infiltration, resultant lipid phagocytosis and exudation, and ultimately, proliferation. The findings of the present study are partially substantiated by a study reporting that ApoE^−/−^ mice on a WD for 10 weeks developed inflammation and emphysema (3). However, the results of the present study contradicted those reported by Samokhin *et al* (4), who claimed that ApoE^−/−^ mice on a high-fat diet developed granulomas. These conflicting results may be partly explained by the difference in the lipid content in the diet and the duration of the high-fat diet. The wild-type mice on the WD exhibited hypercholesterolemia and hypertriglyceridemia with no evidence of lung inflammation and lipidosis. Gene-diet interaction effects were possibly involved in this outcome (19). In the wild-type mice, lipotoxicity induced by the WD resulted in microinflammation only. However, the ApoE-deficient mice treated with the same diet exhibited obvious inflammation injury, suggesting that lipotoxicity or ApoE deletion alone is not sufficient to induce inflammation injury.

### Pulmonary lipidosis in the ApoE^−/−^ WD mice

The primary function of ApoE is to facilitate lipid transport into cells by receptor-mediated endocytosis mediated by the low-density lipoprotein receptor. Adenosine triphosphatase-binding cassette transporter A1 (ABCA1) mediates the efflux of cholesterol to lipid-poor apolipoproteins (ApoA1 and ApoE) (20). It was reported that ABCA1^−/−^ mice displayed lung cholesterol accumulation and inflammation (21). The results of the present study revealed that lung lipidosis occurred in the ApoE^−/−^ mice receiving the WD for 12 weeks. Lipid-laden macrophages were scarce in the ApoE^−/−^ WD mice at 24 weeks, which indicated that the interaction between the genetic and nongenetic factors occurred only at the critical periods.

Several pulmonary diseases are associated with a disruption of lipid homeostasis and inflammation, including endogenous lipid pneumonia, alveolar proteinosis, respiratory distress syndrome and Niemann Pick disease (22). The disease type of the lung abnormity in ApoE^−/−^ WD mice in the present study remains inconclusive.

### TLR4 and activation of downstream molecules in ApoE^−/−^ WD mice

TLR4 downstream signalling comprises at least two distinct pathways as follows: The MyD88-dependent activation of the NF-κB pathway that leads to the production of inflammatory cytokines and a MyD88-independent pathway associated with the production of interferon-beta and the maturation of dendritic cells. The results of the present study showed that TLR4 signalling activated the MyD88/NF-κB and the TRIF/IRF3 pathway to elicit lung inflammation and lipidosis in the ApoE^−/−^ WD mice. It was indicated that MyD88 and p-NF-κB were elevated in the wild-type mice fed a WD, which was partially consistent with a study reporting that a high-fat diet led to the upregulation of TLR4 and NF-κB expression in the intestines of wild-type mice (23).

The present study demonstrated that ApoE deficiency in combination with a WD induces lipidosis and chronic inflammation in lungs through the TLR4 pathway. There is evidence for the correlation between respiratory and cardiovascular diseases (24); however, the precise mechanisms underlying this co-morbidity have remained elusive, and research on the correlation of the two disorders is in its early stages. It is well-documented that TLR4 signalling has an important role in atherosclerosis (25). The findings of the present study illustrated that TLR4 signalling may be a possible common pathway that contributes to lung injury and atherosclerosis, which may provide valuable information for elucidating the lung-heart cross-talk. However, evidence provided in the present study is limited, and future studies on the gene silencing of MyD88 may be required to validate the involvement of TLR4 downstream signaling in the WD-induced lung pathology in the absence of ApoE more convincingly. Due to the complexity of the mechanism of the gene-environment interaction (26), the neuroendocrine system, the changes in the gene methylation pattern and the roles of other TLRs in the animal model used in the present study, further investigation is warranted.

## Figures and Tables

**Table I:** Lipid profile of the ApoE^−/−^ and wild-type mice fed on the high-fat Western-type or normal chow diet for 12 weeks.

Lipid profile	ApoE^−/−^		Wild-type	
	High-fat Western-type	Normal chow	High-fat Western-type	Normal chow
TC (mmol/l)	20.01±3.26^a^,^b^	11.45±0.93^b^	3.25±0.48^a^	2.09±0.47
TG (mmol/l)	2.84±0.35^a^,^b^	1.65±0.54^b^	1.03±0.24^a^	0.81±0.12
Non-HDL (mmol/l)	18.83±0.41^a^,^b^	10.8±2.84^b^	1.71±0.37^a^	0.38±0.55
HDL-C (mmol/l)	0.32±0.23^a^,^b^	0.47±0.16^b^	1.47±0.54	1.59±0.28

Values are expressed as the mean ± standard error.

a P<0.05 compared with mice of the same genotype exposed to the normal diet.

b P<0.05 compared with the wid-type mice fed on the corresponding diet. TC, total cholesterol; TG, triglycerides; HDL-C, high-density lipoprotein cholesterol.
